# Comparison of Decision Tree and Long Short-Term Memory Approaches for Automated Foot Strike Detection in Lower Extremity Amputee Populations

**DOI:** 10.3390/s21216974

**Published:** 2021-10-21

**Authors:** Pascale Juneau, Natalie Baddour, Helena Burger, Andrej Bavec, Edward D. Lemaire

**Affiliations:** 1 Ottawa Hospital Research Institute, Ottawa, ON K1Y 4E9, Canada; pjune022@uottawa.ca (P.J.); elemaire@ohri.ca (E.D.L.); 2 Department of Mechanical Engineering, University of Ottawa, Ottawa, ON K1N 6N5, Canada; 3 University Rehabilitation Institute, University of Ljubljana, 1000 Ljubljana, Slovenia; helena.burger@ir-rs.si (H.B.); andrej.bavec@ir-rs.si (A.B.); 4 Faculty of Medicine, University of Ljubljana, 1000 Ljubljana, Slovenia; 5 Faculty of Medicine, University of Ottawa, Ottawa, ON K1N 6N5, Canada

**Keywords:** 6MWT, foot strike detection, amputee, stride parameters, machine learning, decision tree, deep learning, LSTM, artificial intelligence, smartphone

## Abstract

Foot strike detection is important when evaluating a person’s gait characteristics. Accelerometer and gyroscope signals from smartphones have been used to train artificial intelligence (AI) models for automated foot strike detection in able-bodied and elderly populations. However, there is limited research on foot strike detection in lower limb amputees, who have a more variable and asymmetric gait. A novel method for automated foot strike detection in lower limb amputees was developed using raw accelerometer and gyroscope signals collected from a smartphone positioned at the posterior pelvis. Raw signals were used to train a decision tree model and long short-term memory (LSTM) model for automated foot strike detection. These models were developed using retrospective data (n = 72) collected with the TOHRC Walk Test app during a 6-min walk test (6MWT). An Android smartphone was placed on a posterior belt for each participant during the 6MWT to collect accelerometer and gyroscope signals at 50 Hz. The best model for foot strike identification was the LSTM with 100 hidden nodes in the LSTM layer, 50 hidden nodes in the dense layer, and a batch size of 64 (99.0% accuracy, 86.4% sensitivity, 99.4% specificity, and 83.7% precision). This research created a novel method for automated foot strike identification in lower extremity amputee populations that is equivalent to manual labelling and accessible for clinical use. Automated foot strike detection is required for stride analysis and to enable other AI applications, such as fall detection.

## 1. Introduction

Foot strike (FS) identification is necessary for human gait evaluation, providing insight into a person’s activity levels, mobility, and gait pattern. For example, foot strikes identify the start and end of a gait cycle and can be used to calculate the step time, stride time, and double support time for each leg. Previously, FS identification was completed by visual analysis with video-tracking systems (Vicon, etc.), ground reaction force analysis with force plates, or 3D accelerometer and gyroscope signal analysis from sensors placed at the foot/ankle or shank. While these methods have been successful for both able-bodied and disease populations [1,2,3], they can be expensive, difficult, and timely to set-up. More recently, 3D signals collected from a smartphone located at the pelvis have provided a more accessible analysis of the movement status [4,5,6]. While these models can identify FS with a high accuracy, the FS identification models were typically based on able-bodied participant data. 

Lower limb amputee populations can present with a high variability and inconsistent walking patterns that put them at a high risk of injury and falls [7]. Instability, an asymmetrical gait, or using a walking aid can make it difficult to automatically detect steps from sensor data. Algorithms for gait phase detection for microprocessor controlled prostheses are frequently trained on input data from able-bodied individuals wearing a single sensor on the thigh, shank, or foot, or on signals from multiple sensors on the body [8]. While this may be effective for research purposes, using a single sensor location would facilitate use in clinical environments, where the time is not available to configure multisensory systems on a patient.

Recently, a rule-based foot strike identification algorithm for lower limb amputees was developed using anterior–posterior (AP) linear acceleration collected from a smartphone affixed to the posterior pelvis during a a six-minute walk test (6MWT) [9]. This approach achieved 87% FS detection accuracy, with error correction. When analyzing lower limb amputee gait data for clinical use, manual FS labelling would be needed to ensure appropriate stride timing. For example, recent research by Daines et al. [10] used smartphone data during a 6MWT and manually labelled FS to predict the fall risk for people with lower limb amputations with 81.3% accuracy. While these results are promising, manual labelling is time-consuming and impractical for clinical use, where immediate results reporting is desirable to support decision making at the point of patient contact.

Artificial intelligence (AI) algorithms have been proposed as an alternative method for gait analysis in populations that have a more variable gait, such as cerebral palsy or Parkinson’s disease [11,12,13,14]. Machine learning is a subset of artificial intelligence where algorithms make predictions by evaluating structured data over time. Machine learning models are simple to build, easy to interpret, and require shorter training times than more complex models. A popular supervised machine learning algorithm is the decision tree. Decision trees classify data based on a set of features for each input. The model splits the data based on feature values and their corresponding class labels by determining the most effective decision boundary. Decision trees have been used to diagnose coronary artery disease [15] and to distinguish healthy tissue from cancerous tissue [16]. These models have also been used to perform logistic regression analysis for gait phase recognition to improve dynamic knee–ankle–foot orthosis control [17].

Deep learning is a more complex subset of machine learning. Deep learning models require more training time, but often provide a higher accuracy [18] because they can perform automated feature extraction and classification concurrently, whereas a feature selection process is required prior to training a machine learning algorithm. Deep learning methods also require less time during testing than machine learning techniques if the data set is large. 

Recurrent neural networks (RNN), a deep learning approach, are a class of artificial neural networks that contain both feed-forward and feedback loops, making it possible to loop relevant information back into the network. RNNs perform well on sequential data, such as handwriting recognition [19], and have been implemented for gait segmentation, recognizing heel-strikes and toe-offs by training on data from in-shoe sensors [20]. Long short-term memory (LSTM) is a popular RNN architecture that, like RNN, has both feed-forward and feedback components, and has the addition of a forget gate that sorts data into short-term and long-term memory cells. This process helps to regulate information flow by determining what data should be remembered and what data can be forgotten, making them ideal for data sets that have gaps between relevant events. For example, there is constant movement during gait but a FS only occurs once every 0.4–0.6 s, depending on the walking speed [21]. Recently, LSTM networks have been trained on smartphone sensor data for human activity recognition [22] and gait cycle detection [23].

The effectiveness of machine learning and deep learning algorithms to classify FS in lower limb amputee populations using smartphone signals has not yet been evaluated or compared. This research developed a novel method for automated FS detection using filtered acceleration and gyroscope signals collected from a smartphone during a 6-min walk test, using both decision tree and LSTM approaches. A viable model will provide the basis for automated stride parameter calculation and stride segmentation, which is essential for using new fall risk and health status AI models within clinical environments.

In this paper, Section 2 details the methodology and experimental design. Section 3 details the results obtained from this research. Section 4 provides a discussion of the results and their implications. Section 5 provides a conclusion and details future research.

## 2. Materials and Methods

Section 2 is structured as follows. Section 2.1 details participant recruitment and characteristics. Section 2.2 describes the experimental setup and data collection process. Section 2.3 discusses pre-processing, with Section 2.3.1 covering signal filtering and processing and Section 2.3.2 detailing the manual labelling of the ground truth foot strikes. Section 2.4 describes the construction of the classification models, where Section 2.4.1 describes the decision tree classifier and Section 2.4.2 describes the LSTM classifier. Section 2.5 details the evaluation metrics for classification. Section 2.6 presents the post-processing error correction.

### 2.1. Recruitment and Participants

A convenience sample of 93 transtibial, transfemoral, and bilateral lower limb amputees were recruited from the University Rehabilitation Institute (Ljubljana, Slovenia). The inclusion criteria were: transtibial or higher amputation; ability to walk with single cane, 2 crutches, or without any walking aids; minimum of 6 months post-amputation; had a functional prosthesis; no wounds on the residual limb; and was willing to participate. Only participants who completed the full 6 min were included in this analysis. Excluded trials were due to incomplete trial (15), cell phone affixed to the side of the hip instead of lower back (5), and use of a non-rolling walker (1). Therefore, 72 participants (14 female, 58 male, age 62.3 ± 12.7) were included in this study. Participants included 63 transtibial, 5 transfemoral, and 4 bilateral transtibial amputees. Ten participants (13.9%) completed the 6MWT with a single cane/crutch, 22 participants (30.6%) walked with double crutches, and 40 participants (55.5%) walked without gait aids. All participants provided informed consent.

### 2.2. Data Collection

**Figure 1:**
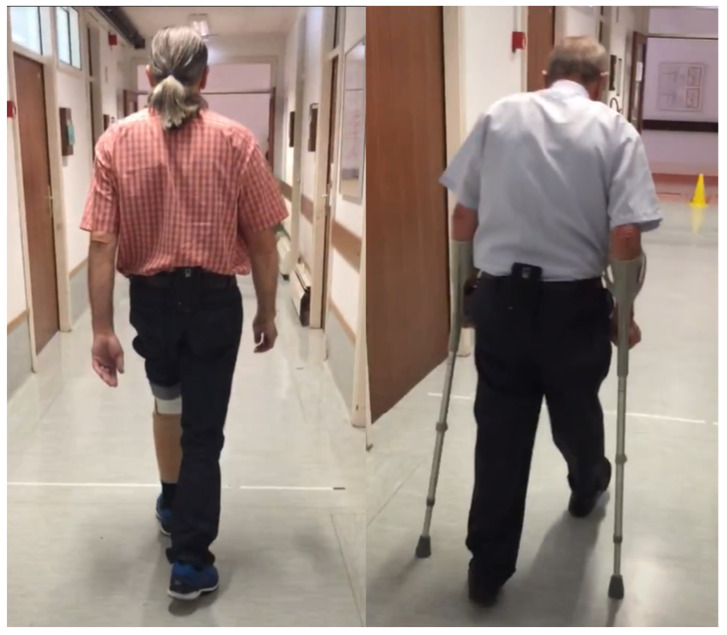
Experimental set-up: smartphone on posterior pelvis.

An Android smartphone was placed on a belt at the lower back of each participant before completing a 6MWT along a 20 m hallway (Figure 1). Accelerometer, gyroscope, and smartphone orientation data were collected with the TOHRC Walk Test app at 50 Hz. Each participant was video recorded for the duration of their 6MWT.

### 2.3. Pre-Processing

#### 2.3.1. Filtering and Signal Processing

Once the test was complete, data were exported from the smartphone for pre-processing. Raw accelerometer data, gyroscope data, smartphone orientation, and timestamps for each recording were imported into MATLAB 2020b. Signals were filtered with a fourth-order zero-lag Butterworth low pass filter with a cut-off frequency of 4 Hz. Smartphone orientation, XYZ coordinates for raw and linear acceleration (m/s^2^), and angular velocity (rads/s) were the input data. Since smartphone signals are collected at a variable sampling rate, each signal was re-interpolated at 50 Hz for a total of 18049 data points per participant over the 6-min walk test.

#### 2.3.2. Manual Ground Truth Labelling

Ground truth steps were manually identified and labelled by two assistants prior to model training as label 0 (no foot strike present) and label 1 (foot strike present) using the following procedure. Linear acceleration signals over time were graphed. In a typical gait cycle, AP acceleration peaks coincide with FS events, followed by a vertical acceleration peak. Therefore, AP signal peaks immediately followed by a vertical signal peak were identified and the timestamp recorded as a FS event. Participant video was used to confirm timestamps. In cases where the AP peak was not well defined (e.g., gait irregularity, instability, etc.), a consensus of the two assistants was made and the most appropriate location was selected. All other timestamps were consequently labelled as “no foot strike present”.

### 2.4. Classification Models

#### 2.4.1. Decision Tree

Models were written and evaluated in Python 4.1. The decision tree classifier and evaluation metrics were imported from scikit-learn library. Training data included the 12 smartphone signals and the corresponding ground truth labels for each data point. Hyperparameters evaluated included maximum tree depth and class weighting (1:2, 1:5, 1:10, 1:20). The default options in the scikit-learn library were used for all other training parameters.

#### 2.4.2. LSTM

The LSTM model was imported from Keras. Smartphone signals were formatted into data windows prior to model input. Each window spanned 15 frames (0.3 s) before the class label to 15 frames after the label. For the first 15 data points, 30 frames after the class label were used. Similarly, the previous 30 frames were used for the final 15 data points. The 31-frame window size (i.e., 15 before, labelled frame, 15 after) minimized the likelihood of more than one FS event occurring within the same window. Several hyperparameter combinations were evaluated, including batch size (32, 64, 128), number of hidden LSTM and hidden dense nodes (25, 50, 75, 100), dropout (0.3, 0.4, 0.5), and class weighting (1:2, 1:5, 1:10, 1:20). Since this was a binary classifier, binary cross-entropy was used as the loss function. Dense layer activation functions included ReLU in the input layer and sigmoid in the output layer. To evaluate the model, a confusion matrix module was imported from the scikit-learn library.

### 2.5. Classifier Evaluation

Five-fold cross validation was used to evaluate performance of both AI models. A temporal tolerance of ±2 frames (±0.04 s) was used to match ground truth manually labelled FS labels with predicted class labels. The results were evaluated based on sensitivity, specificity, accuracy, and precision.

Stride parameters were calculated using both manually labelled ground truth and predicted FS. The difference between these ground truths and predicted values for step time, stride time, and cadence were compared to the minimal detectable change (MDC) for each value. Since MDC was not available for lower limb amputee gait, MDC of stride parameters for healthy older adults was used [24,25,26].

### 2.6. Post-Processing

**Figure 2:**
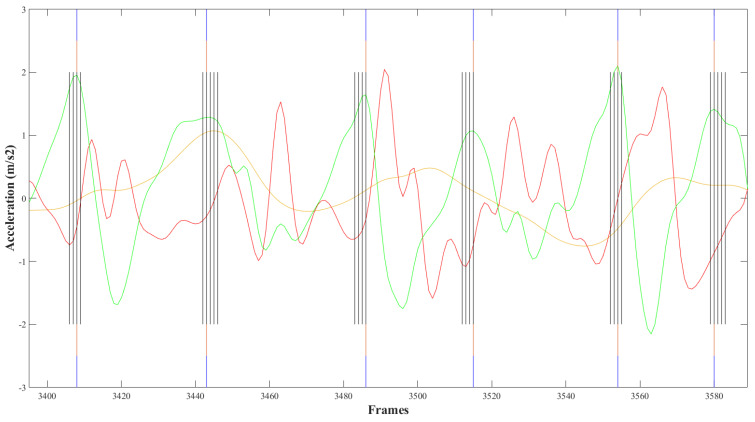
“Banded” groupings of foot strike predictions surrounding AP signal peak occurred in all participants. Anterior-posterior (green), vertical (red), and medio-lateral (yellow) accelerations for 6MWT were graphed over time. Black vertical lines indicate model predictions of “FS present” prior to correction. Blue lines indicate ground truth labels for foot strikes. Orange lines indicate adjusted predictions corresponding with a peak in AP acceleration with banded periods.

FS predictions and linear acceleration signals over time were graphed to evaluate preliminary model performance. Typically, a single FS event would correspond with the AP signal peak. However, upon visual examination of the initial test results, periods of multiple consecutive predictions of the “FS present” class label corresponding to a single AP peak were observed. The predictions were often located prior to, at the FS instance, and immediately following the AP signal peak, causing a “banded” appearance on the graph. In order to correct for this “banding”, instances where two or more FS classifications occurred consecutively were identified in MATLAB. The timestamps of the start and end of each period of multiple FS and the corresponding AP acceleration signal for this period were recorded. The peak AP acceleration within the period was identified. The FS label at this timestamp replaced all other FS labels in that period (Figure 2).

To correct for missed steps, a method similar to Capela et al. [4] was employed. A locking period specific to each participant’s trial was defined from a 5-s sample of the filtered vertical acceleration signal from the beginning of the 6MWT trial. The time between positive zero-crossings for the vertical acceleration signal in the sample was used to calculate the locking period based on three procedures:

- The default locking period was half the maximum time between zero crossings;
- If the maximum time between zero crossings was greater than 0.6 s, the locking period was half the mean time between zero crossings;
- If the maximum time between zero crossings was less than 0.3 s, the maximum time between zero crossings was multiplied by 2.

**Figure 3:**
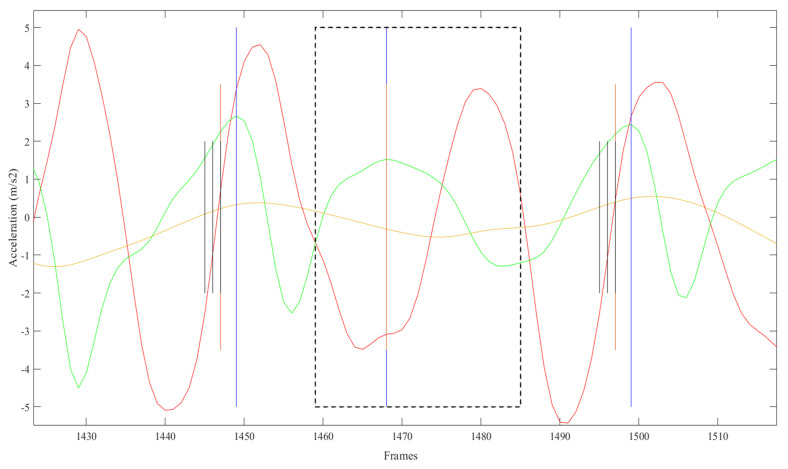
Missed step (vertical blue line) identified within adjusted search range (black dotted line). FS inserted (vertical orange line) at timestamp for the peak AP acceleration (green line) in this period.

To identify missed steps, periods where the duration between two consecutive steps was greater than 1.5 times the previous step were identified. The start of the period was increased by half the locking period, and end of the period was decreased by the same amount (i.e., so that the missed step was not inappropriately located at the start or end of the original selected period). FS was inserted at the timestamp for the peak AP acceleration in this period (Figure 3).

## 3. Results

A total of 39,561 foot strikes were identified and labelled in the ground truth data, accounting for 3.04% of total output labels (1,299,528). Table 1 displays confusion matrices for the decision tree and LSTM models. The best performing decision tree model had a maximum tree depth of 10 and class weighting of 1:20 (label 0: label 1). The decision tree classification accuracy was 98.7%, sensitivity was 82.8%, specificity was 99.2%, and precision was 78.6%. The LSTM model with the best performance had a batch size of 64, dropout of 0.4, one LSTM layer with 100 hidden LSTM nodes, one dense layer with 50 hidden dense nodes, and a class weighting of 1:2 (label 0: label 1). The LSTM classification accuracy was 99.0%, sensitivity was 86.4%, specificity was 99.4%, and precision was 83.7%.

Differences in stride parameter outcome measures between manual and automated FS for each model are displayed in Table 2. The step time and stride time differences were within the MDC for both models, whereas the differences in cadence were outside the MDC for both models. The LSTM model had smaller differences overall.

The automated band and missed step corrections were essential (Table 3). The LSTM results improved by 8.2% for sensitivity, 3.7% for specificity, 3.9% for accuracy, and, the most notable increase, 61.9% for precision.

**Figure 4:**
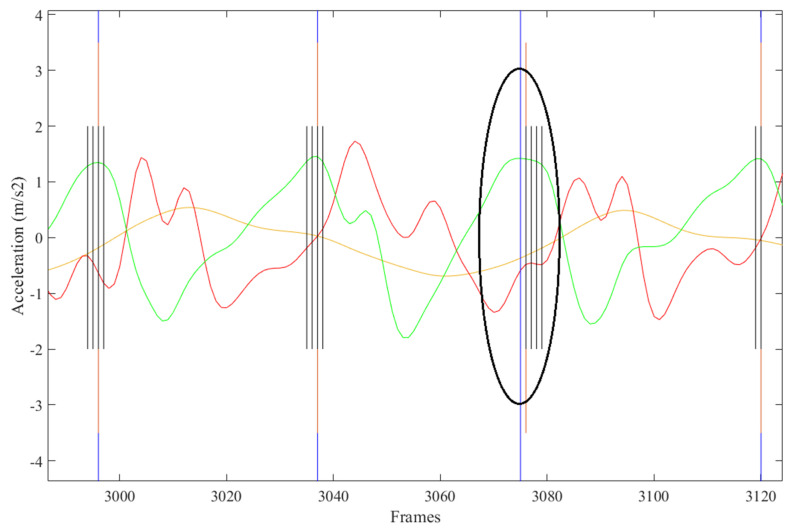
Automated FS (vertical orange line) inserted within five frames of manually labelled FS (vertical blue line).

**Figure 5:**
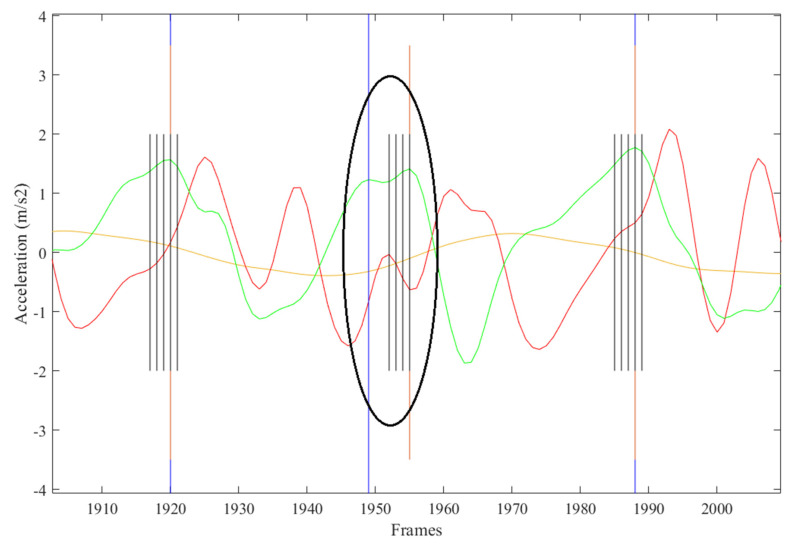
Automated FS inserted more than five frames from manually labelled FS.

**Figure 6:**
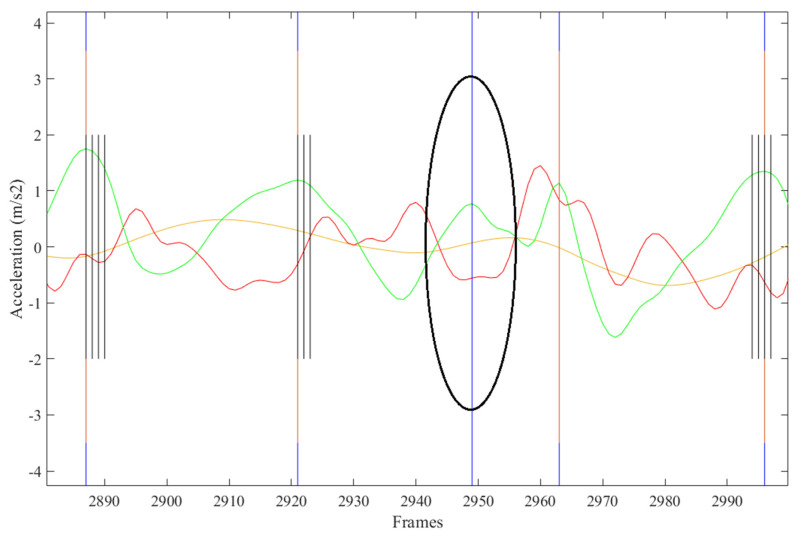
Manually labelled FS not identified by AI model.

**Figure 7:**
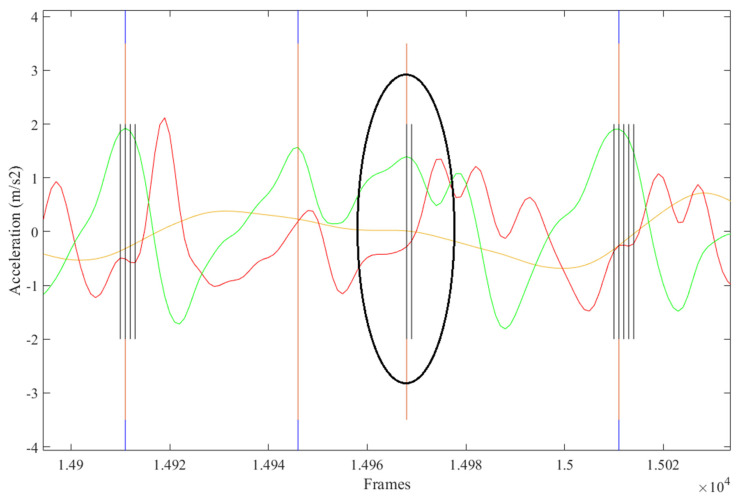
Extra FS inserted by AI model.

The FS identification error was 13.6%. Contributions to this error rate included automated FS labelled within +/− five frames of manually labelled FS (Figure 4), automated FS greater than five frames from manually labelled FS (Figure 5), steps missed by the AI not corrected for (Figure 6), and extra steps inserted by the AI model (Figure 7).

## 4. Discussion

This research successfully created an automated foot strike detection model that only requires smartphone acceleration, angular velocity, and orientation data from a posterior pelvis location. The LSTM model outperformed the decision tree in all areas of analysis and is the recommended model for future applications. 

Compared to the previous rule-based lower limb amputee FS detection model in [9], the LSTM resulted in an improved FS classification. The algorithm described in [9] performed FS identification by using either AP acceleration or vertical acceleration; whichever provided a smoother signal. The LSTM model was trained on all 12 signals collected during the 6MWT. The inclusion of additional signals may have resulted in a more accurate FS identification by identifying patterns in all signals where a FS occurred. In addition, the increased complexity of the LSTM architecture may have been better suited to lower limb amputee gait variability.

Previous research [10] demonstrated that clinically relevant outcomes, such as fall risk, can be identified in amputees using 6MWT data and a random forest model. However, automated stride detection would be required to enable the system to automatically run the model, since data features are calculated for each stride. Implementation on a smartphone would allow any clinician to complete a 6MWT assessment and view the stride parameter outcome measures and fall risk status immediately after completing the trial (i.e., instant reporting). 

The LSTM model had a FS prediction error rate of 13.6%. When the temporal tolerance was adjusted from ± two frames to ± five frames, as employed in Tan et al. [23], the FS prediction error decreased to 12.6%, showing that a small percentage of errors were within five frames (0.1 s) of the manually labelled FS. Interestingly, in some cases where the automated FS was predicted to be more than five frames from the manually labelled FS, the AI model may have selected a more appropriate peak. For example, in Figure 5, a double peak is visible in the AP acceleration signal (green line). The manually labelled FS at frame 1949 corresponds with the first peak and the automatically labelled FS at frame 1955 corresponds with the second and greater peak. Looking at the previous step and the following step, the manually labelled FS corresponds with an AP acceleration peak immediately prior to a vertical acceleration peak (red). Given this pattern in acceleration signals prior to and after this timepoint, the predicted FS at frame 1955 is likely to be a more appropriate placement than at frame 1949.

Other errors included manually labelled steps that were not identified by the LSTM model that were not corrected for later, and extra FS inserted in an inappropriate location. The use of walking aids, such as canes or crutches, can cause double peaks or abnormally-shaped curves in the acceleration signal, which can lead to these FS identification errors. In addition, steps that occur during a period of instability or if the person is walking asymmetrically (which is common for lower limb amputees) can cause similar errors. Another factor contributing to the error rate could be errors in manually identifying the ground truth foot strike events, where the visual identification of a foot strike could be off by one frame. 

The error rate did not adversely affect the clinical outcome measures, where the difference between the automated and manually labelled FS step time and stride time was within the MDC. The difference in cadence was outside the MDC for both LSTM and decision tree models. However, the MDC for these parameters was not available for lower limb amputees; instead, values were compared with healthy older adult MDC. This suggests that, when extracting stride parameters from the 6MWT in lower limb amputees, clinical outcome measures from the automated FS are equivalent to measures calculated from manually labelled events.

For both models, postprocessing to select one event within “banded” predictions was necessary to improve the model performance. Repetitive series of FS predictions surrounding the manually labelled ground truth FS resulted in a greater number of false positives and fewer true negatives, affecting all classification results, and, in particular, there was a notable decrease in precision (Table 3). Band correction was very effective in automatically selecting the appropriate acceleration peak, but would only be viable in post-processing, and not for real-time FS detection. 

This research had several limitations. Only those who completed the full 6MWT were included in this research. While stopping to rest during the 6MWT is permitted, the inability to complete the full 6 min is an indication of impairment that could be clinically relevant. As such, excluding them from the training data could limit model generalizability, reducing the accuracy for patients of decreased ability levels. A larger subset of people not completing the 6MWT would be required to improve the model. In addition, while participants with canes and crutches were included, those using non-rolling walkers were excluded from this analysis. Further subgroup analyses should be completed to investigate if the current model is also applicable to these groups. Since the study population sample only included five people with transfemoral amputation and four with bilateral amputation, further research could be performed to determine if the model would improve with more participants with these characteristics in the training set.

## 5. Conclusions

FS identification is essential to define the gait cycle and calculate stride parameters. AI tools for clinical analysis (e.g., fall risk classification) rely on proper gait segmentation to calculate step-based features. In lower limb amputees, manual step identification was required due to the high gait variability and irregularity, limiting the clinical viability of such tools in this population [10]. This research developed a novel LSTM approach for automated FS detection in lower limb amputee populations using smartphone sensor signals at the posterior pelvis. A LSTM deep learning model was more effective for FS identification in lower limb amputees than a decision tree machine learning model. Post-processing further improved the classification results. Stride parameters calculated using predicted FS were equivalent to those calculated from manually labelled FS, demonstrating that the automated FS with smartphone sensor data could be viable for clinical analysis. Future research could include a sub-group analysis of participants who did not complete the full 6MWT and those using mobility aids, such as wheeled walkers, since using these aids can provide signal characteristics that confuse AI classifiers. Additionally, this model could be validated for use in other disability groups, such as Parkinson’s disease or cerebral palsy. The implementation of this FS detection model on a smartphone, as an improvement to the TOHRC Walk Test app, for example, would bring the advancements from this research to daily clinical use and improve clinical decision making for the lower limb amputee population.

## Figures and Tables

**Table 1:** Confusion matrices.

Decision Tree			LSTM		
	Foot Strike	No Foot Strike		Foot Strike	No foot Strike
Foot strike	32,849	6712	Foot strike	34,200	5361
No foot strike	10,410	1,244,508	No foot strike	7165	1,246,603

**Table 2:** Average and standard deviation (in brackets) difference between manual and automated foot strike stride parameter outcome measures for LSTM and decision tree (DT) models. MDC = minimum detectable change.

	LSTM	DT	MDC
Step time (s)	0.0010 (0.29)	−0.0139 (0.22)	0.042
Stride time (s)	−0.0006 (0.26)	−0.0149 (0.20)	0.772
Cadence (steps/min)	29.47 (39.87)	56.04 (53.35)	8.44

**Table 3:** Evaluation metrics before and after automated corrections.

	Sensitivity	Specificity	Accuracy	Precision
After correction	86.4%	99.4%	99.0%	83.7%
Before correction	78.2%	95.7%	95.1%	21.8%

## Data Availability

Not applicable.
